# Association Study of Hyaluronan-Binding Protein 2 (*HABP2*) Gene Polymorphisms in Idiopathic Recurrent Pregnancy Loss (RPL) in Korean Women

**DOI:** 10.3390/ijms262411813

**Published:** 2025-12-07

**Authors:** Jeong Yong Lee, Young Ran Kim, Eun Ju Ko, Hyeon Woo Park, Jae Hyun Lee, Seung Ho Hong, Ji Eun Shin, Eun Hee Ahn, Ji Hyang Kim, Nam Keun Kim

**Affiliations:** 1 Department of Life Science, Graduate School, CHA University, 335 Pangyo-ro, Seongnam 13488, Republic of Korea; smilee3625@naver.com (J.Y.L.);; 2 Division of Life Sciences, College of Life Sciences, CHA University, 335 Pangyo-ro, Seongnam 13488, Republic of Korea; 3 Department of Obstetrics and Gynecology, CHA Bundang Medical Center, School of Medicine, CHA University, 59 Yatap-ro, Seongnam 13496, Republic of Korea; happyiran@cha.ac.kr (Y.R.K.);; 4 Department of Science Education, Teachers College, Jeju National University, 61 Iljudong-ro, Jeju 63294, Republic of Korea

**Keywords:** single nucleotide polymorphisms, infertility, factor VII-activating protease (*FSAP*)

## Abstract

Recurrent pregnancy loss (RPL), also termed recurrent spontaneous abortion, is defined as the failure of ≥2 consecutive pregnancies before 20 weeks of gestation. Approximately 5% of pregnant couples experience RPL. The hyaluronan-binding protein 2 (*HABP2*) gene is involved in coagulation and plays an important role during pregnancy. In >50% of RPLs, the etiology remains unexplained. We collected 765 blood samples from 388 female RPL patients and 377 healthy female controls. To investigate the relationships between *HABP2* polymorphisms and RPL, we examined six *HABP2* variants (rs3832698 A>del, rs10885478 G>A, rs932650 T>C, rs7923349 G>T, rs1157916 G>A, and rs2240879 T>C) to clarify their association with RPL risk. The rs2240879 CC genotype was significantly associated with an increased RPL risk (*p* = 0.028). In haplotype analysis, the combination of rs3832698 del and rs2240879 T (del-T) was associated with elevated risk (*p* = 0.043); this risk persisted in combinations with additional polymorphisms (rs3832698 A>del, rs10885478 G>A, rs932650 T>C, rs7923349 G>T; del-A-T-T, *p* < 0.001; rs3832698 A>del, rs10885478 G>A, rs932650 T>C, rs7923349 G>T, rs1157916 G>A, rs2240879 T>C; del-A-C-T-G-T, *p* = 0.024). The rs3832698 and rs1157916 genotypes were significantly associated with prothrombin time (*p* = 0.020 and *p* = 0.012, respectively). We identified associations between *HABP2* polymorphisms and RPL; rs2240879 was linked to an increased RPL risk. Additionally, rs3832698 was associated with an altered prothrombin time. These findings suggest that *HABP2* represents a biomarker for RPL susceptibility.

## 1. Introduction

Recurrent pregnancy loss (RPL), also termed recurrent spontaneous abortion, is defined as the failure of ≥2 consecutive pregnancies before 20 weeks of gestation [1,2]. Approximately 5% of pregnant couples experience RPL [1,3,4]. Multiple factors contribute to RPL, including chromosomal abnormalities, uterine anomalies, infections, hormonal imbalances, immune dysfunction, and environmental conditions. However, in >50% of couples diagnosed with RPL, the etiology remains unexplained.

Recent studies have detected associations between single nucleotide polymorphisms (SNPs) and the pathogenesis of various diseases, including RPL [5,6,7]. SNPs serve as valuable genetic markers for investigating interindividual differences in disease susceptibility and drug responses, including those related to solid tumors, coronary artery disease, and RPL [8,9,10].

In a previous study, we identified the hyaluronan-binding protein 2 (*HABP2*) gene through whole-exome sequencing in cases of RPL [11]. The *HABP2* gene, located on chromosome 10, contains 13 exons [12]; it is also known as Factor VII-activating protease (*FSAP*). Factor VII is a component of the tissue factor (extrinsic) coagulation pathway, which plays a critical role during pregnancy [13]. *HABP2* encodes an extracellular serine protease that binds to hyaluronic acid, a glycosaminoglycan abundantly present in the female reproductive tract. Hyaluronic acid, a key component of the extracellular matrix, promotes angiogenesis [14,15]. Multiple studies have investigated genetic variations in *HABP2*, particularly concerning two nonsynonymous polymorphisms in the coding region. One of these, Gly534Glu (Marburg I), substantially reduces the proteolytic activity of HABP2. The other, Glu393Gln (Marburg II), has not been linked to changes in enzymatic function or other protein characteristics [16]. *HABP2* variants have been associated with pregnancy-related conditions, including RPL [17,18,19].

In this study, we analyzed the relationships between *HABP2* polymorphisms and RPL. Six polymorphisms (rs3832698 A>del, rs10885478 G>A, rs932650 T>C, rs7923349 G>T, rs1157916 G>A, and rs2240879 T>C) located in the promoter region, introns, and 3′ untranslated region (UTR) were selected; however, even non-coding regions can possiblly change gene expression [20,21,22,23]. Therefore, we examined their frequencies in Korean women with RPL and in healthy controls.

## 2. Results

### 2.1. Characteristics of the Study Population

The baseline characteristics of the RPL and control groups are presented in Table 1. No significant difference in age distribution was observed between the groups, indicating that frequency matching by age and sex was successful. RPL patients exhibited a significantly lower prothrombin time (PT; *p* < 0.001) and activated partial thromboplastin time (aPTT; *p* < 0.001) compared with controls. However, platelet levels, another important coagulation parameter, did not significantly differ between the groups. Significant differences were observed in FSH, estradiol (E2), and TSH levels between the RPL and control groups; LH levels did not significantly differ. Additionally, no significant differences were observed in folate (*p* = 0.971) or homocysteine (*p* = 0.845) levels between the two groups.

### 2.2. Genotype Analysis of RPL and Control Groups

The distribution of *HABP2* polymorphisms—rs3832698 A>del, rs10885478 G>A, rs932650 T>C, rs7923349 G>T, rs1157916 G>A, and rs2240879 T>C—was examined in RPL patients and control participants (Table 2). Adjusted odds ratios (AORs) were calculated using logistic regression, with age as a covariate. Several polymorphisms showed significant differences between the RPL and control groups. The rs2240879 T>C variant demonstrated a significant association with RPL (TT vs. CC: crude OR, 3.365; 95% CI, 1.232–9.196; AOR, 3.424; 95% CI, 1.252–9.369). In the subgroup analysis of patients with >3 pregnancy losses, the rs3832698 A>del variant showed a significant association (AA vs. A del: AOR, 0.253; 95% CI, 0.075–0.863; *p* = 0.039), as did the rs7923349 variant under the recessive model (AOR, 0.112; 95% CI, 0.018–0.700; *p* = 0.046).

### 2.3. Genotype Combination Analysis of Polymorphisms with Synergistic Effects

To investigate genetic effects in the absence of environmental influences, we analyzed the combined effects of *HABP2* genotypes on RPL prevalence (Table 3). Significant associations were identified for the combination of rs7923349 G>T and rs2240879 T>C (GG-CC: AOR, 3.066; 95% CI, 0.801–11.734; *p* = 0.043; GT-CC: AOR, 6.869; 95% CI, 0.8269–57.0576; *p* = 0.025). Additionally, the combination of rs2240879 T>C and rs1157916 G>A was associated with an increased RPL risk (TT-AA: AOR, 3.310; 95% CI, 1.192–9.193; *p* = 0.038).

### 2.4. Haplotype Analysis of Polymorphisms with Synergistic Effects

To further evaluate genetic contributions independent of environmental factors, we performed a haplotype analysis of *HABP2* alleles (Table 4). A significant association was observed for the combination of rs3832698 A>del and rs2240879 T>C (del-T: OR, 1.231; 95% CI, 0.972–1.559). In the three-SNP analysis, significant associations were identified for the rs3832698 A>del/rs10885478 G>A/rs2240879 T>C haplotype (del-G-T: OR, 1.327; 95% CI, 1.020–1.726) and for the rs3832698 A>del/rs932650 T>C/rs2240879 T>C haplotype (A-T-T: OR, 1.229; 95% CI, 0.964–1.567). Haplotypes involving the rs3832698 A>del/rs2240879 T>C combination remained significantly associated with RPL in extended models. In the four-SNP haplotype (rs3832698 A>del/rs10885478 G>A/rs932650 T>C/rs7923349 G>T), the del-A-T-T haplotype showed a strong association (*p* <0.001). Similarly, the five-SNP haplotype rs3832698 A>del/rs10885478 G>A/rs932650 T>C/rs7923349 G>T/rs2240879 T>C (del-A-C-T-T) was significantly associated with RPL (*p* = 0.030).

### 2.5. Interaction Analysis Between Genes and Clinical Factors

**Figure 1:**
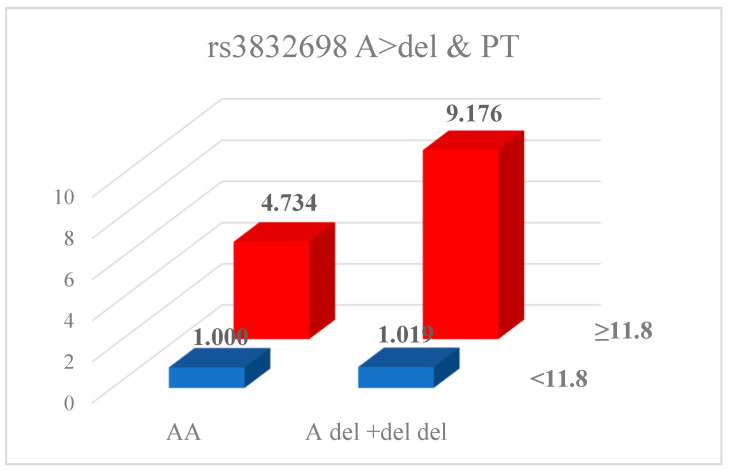
Synergic effect of rs3832698 A>del and PT.

**Figure 2:**
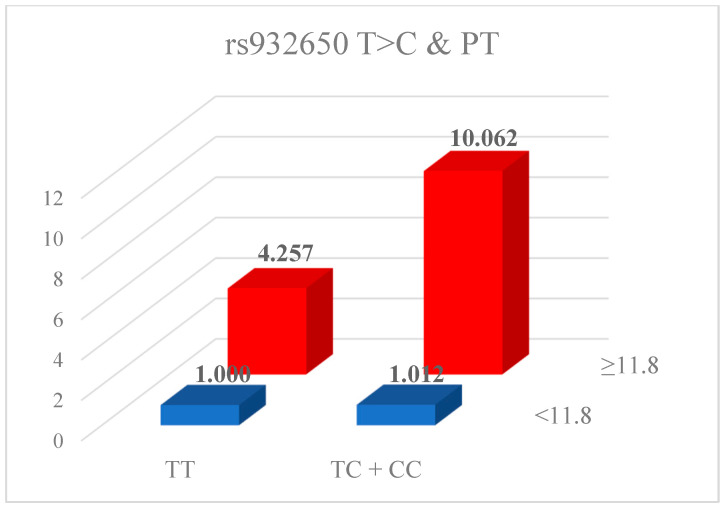
Synergic effect analysis for interplay between clinical factors of rs932650 T>C and PT.

We analyzed the potential synergistic effects between genetic variants and clinical factors, including hormone and coagulation indicators such as PT, aPTT, FSH, and E2 (Table 5). The rs3832698 polymorphism demonstrated synergistic interactions with E2 and LH (rs3832698 A/del + del/del with E2: AOR = 4.762; 95% CI = 2.133–10.630; rs3832698 AA with LH: AOR = 0.210; 95% CI = 0.101–0.440). The *HABP2* rs10885478 GA + AA genotype showed significant associations when combined with TSH (AOR = 7.108; 95% CI = 2.069–24.421) and E2 (AOR = 4.659; 95% CI = 1.314–16.521). The rs1157916 GA + AA genotype also exhibited a significant interaction with E2 (AOR = 4.685; 95% CI = 1.537–14.283). All six investigated SNPs were significantly associated with coagulation indicators PT and aPTT; however, no associations were observed with platelet levels. Figure 1 and Figure 2 illustrate the synergistic effects between PT and either the rs932650 polymorphism or rs3832698 polymorphism.

### 2.6. One-Way Analysis of Variance Between Clinical Parameters and HABP2 Polymorphisms

**Figure 3:**
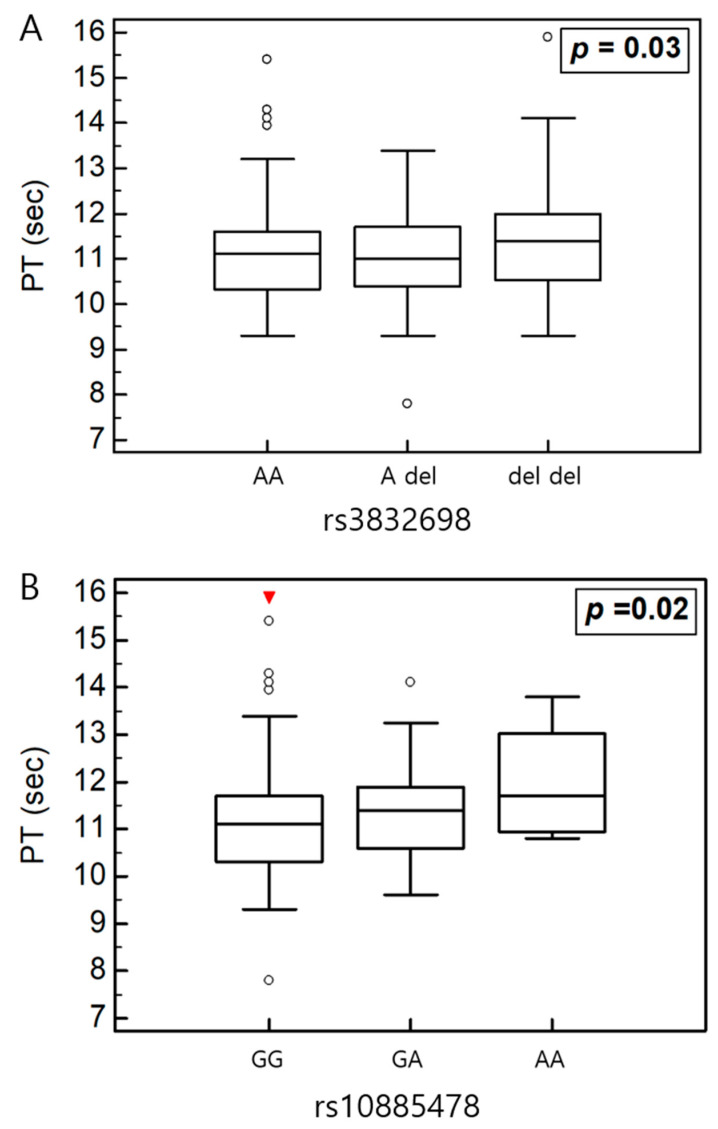
(**A**) One-way analysis of variance in rs3832698 and PT. (**B**) One-way analysis of variance in rs108854798 and PT.

**Figure 4:**
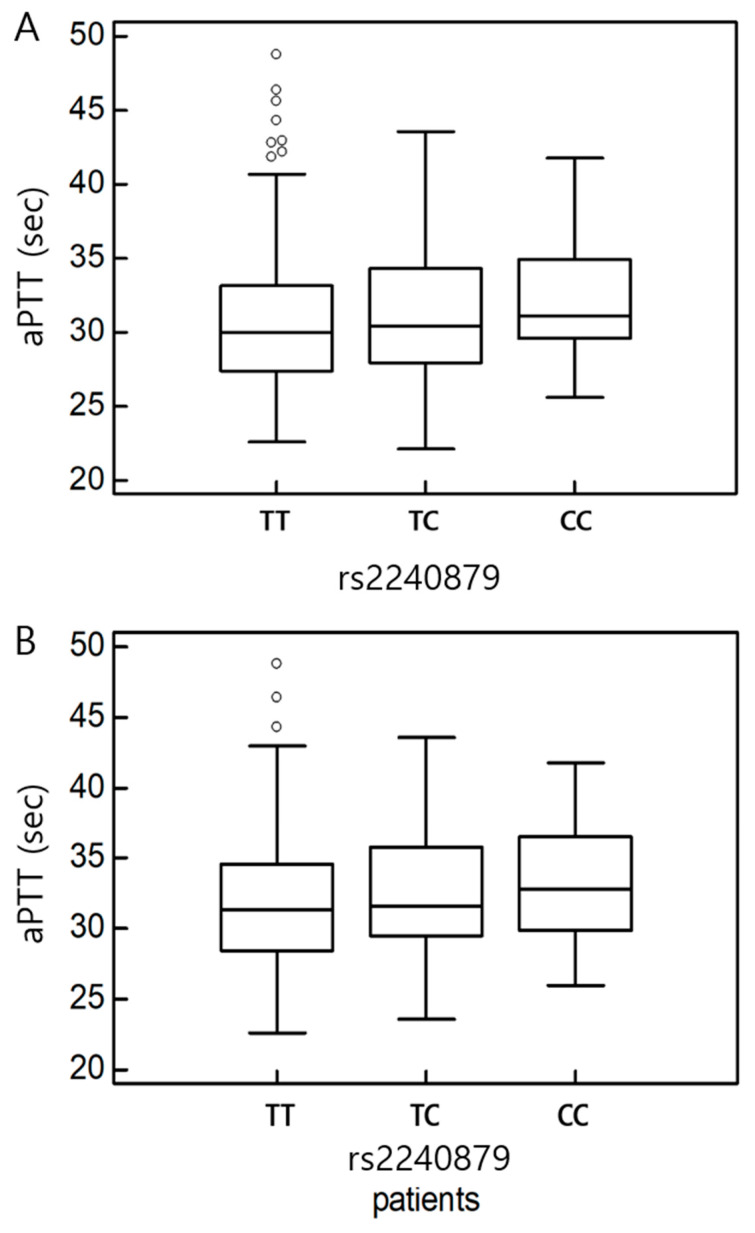
(**A**) One-way analysis of variance in rs2240879 and aPTT in total participants. (**B**) One-way analysis of variance in rs2240879 and aPTT in patients.

**Figure 5:**
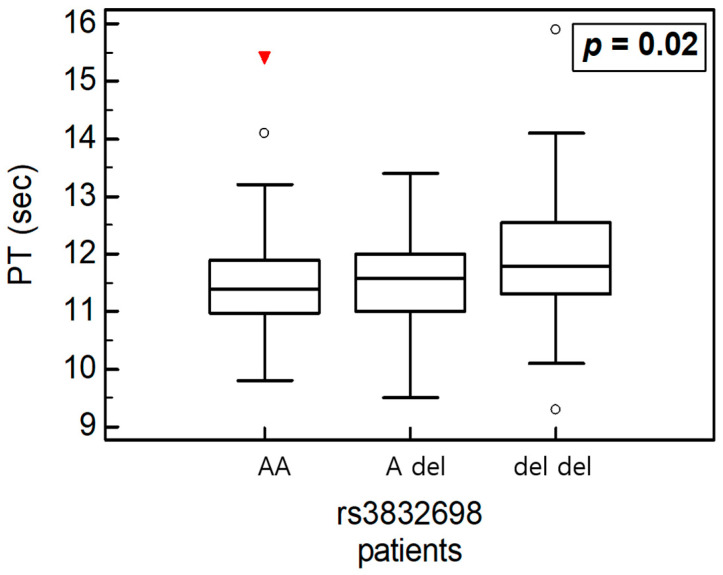
One-way analysis of variance in rs3832698 and PT in patients.

**Figure 6:**
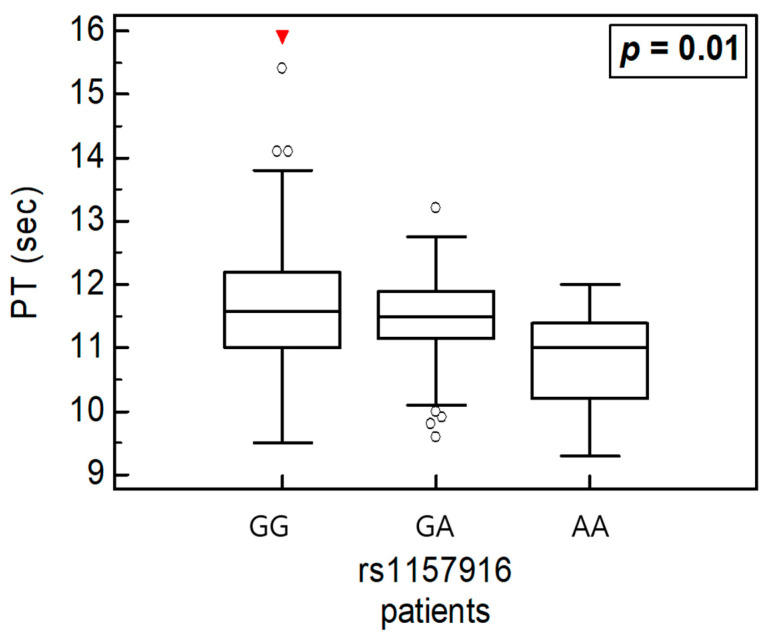
One-way analysis of variance in rs1157916 and PT in patients.

We conducted a variance analysis to examine the associations between *HABP2* polymorphisms and clinical parameters in all participants and subgroup analyses (Table 6, Table 7 and Table 8). In the overall cohort, the PT level was significantly associated with rs3832698 A>del and rs10885478 G>A polymorphisms (*p* = 0.031 and *p* = 0.029, respectively; Figure 3). The aPTT level tended to increase in the presence of the rs2240879 T>C genotype (Figure 4). In the patient subgroup, rs3832698 A>del was associated with a progressive increase in PT level according to genotype (Table 7; Figure 5). Additionally, rs1157916 T>C was significantly associated with variations in the PT level according to genotype within the patient group (Figure 6).

## 3. Discussion

In this study, we selected the *HABP2* gene based on whole-exome sequencing data and then genotyped six polymorphisms. Each polymorphism is well established and matched with Korean minor allele frequencies, such as KRGDB and Korea 1K. And all SNPs are satisfied with the Hardy–Weinberg equilibrium in the control group. In contrast, several SNPs showed a deviation from HWE in patient groups, as HWE disruption in affected individuals may reflect an associated disease or the selection of selection pressure. Therefore, the HWE deviation in patients does not undermine the allele frequency comparison or the conclusions of the study.

Among these, rs2240879 exhibited a significant difference in distribution between RPL patients and controls. This variant is located in the 5′ UTR, which may influence promoter activity and affect protein expression [24]. In patients with >3 pregnancy losses, rs7923349 and rs3832698 showed protective associations. The rs3832698 variant, located in the 3′ UTR, may also influence protein expression through regulatory mechanisms similar to those affected by rs2240879. The haplotype analysis further supported a significant association involving the rs3832698 A>del and rs2240879 T>C combination (Table 4). The del-T haplotype (i.e., the rs3832698 variant and major allele of rs2240879) remained significantly associated with RPL risk when additional *HABP2* polymorphisms were included. These findings were consistent with the results of individual genotype analyses, suggesting that the two UTR-located variants (3′ and 5′, respectively) jointly contribute to RPL susceptibility. In the analysis of gene–clinical parameter interactions, PT and aPTT demonstrated synergistic effects with *HABP2* polymorphisms. HABP2, also known as FSAP, plays a role in the coagulation cascade. However, platelet levels did not show significant associations with any tested variants. The analysis of variance (Table 6, Table 7 and Table 8) revealed that rs3832698 and rs10885478 were significantly associated with the PT level (*p* = 0.031 and *p* = 0.029, respectively). In the subgroup of RPL patients, platelet levels significantly differed according to the rs932650 T>C polymorphism. PT levels were also influenced by the minor alleles of rs3832698 and rs1157916. PT levels tended to increase in relation to the rs10885478 G>A minor allele. Notably, rs2240879 T>C was linked to increases in both PT and aPTT according to the minor allele dosage. These trends were observed in both the total cohort and RPL subgroup; they were not evident in the control group.

HABP2, also known as FSAP, can directly activate Factor VII, and is also referred to as a urokinase-type plasminogen activator [25]. Factor VII is a key component of the coagulation cascade, particularly within the tissue factor pathway, which represents the initial step of the extrinsic coagulation pathway. In this process, Factor VII is converted to its active form, Factor VIIa. FSAP activity is inhibited by tissue factor pathway inhibitors [26]. According to Riondino et al. [19], FSAP levels are higher in women, and the synthesis of FSAP is associated with female sex hormones. This finding aligns with our observation of significant interactions between E2 levels and specific polymorphisms, as shown in Table 6. Accordingly, FSAP expression is influenced by oral hormonal contraceptive therapy [19]. During pregnancy, the levels of nearly all coagulation factors increase; Factor VII increases by up to 1000% [13]. This elevation leads to a shortening of PT. Our results, including synergistic analyses and analysis of variance findings (Table 6, Table 7 and Table 8), are consistent with these physiological changes. FSAP exists in two structural forms: a single-chain form (scFSAP), which functions as a zymogen, and a heterodimeric two-chain form (tcFSAP), which represents the active form [27,28]. Multiple studies have explored how scFSAP is converted into tcFSAP [29,30]. The structure of tcFSAP suggests that the chains bind together regardless of their activation state; however, the precise mechanism remains unclear. Yamamichi et al. [31] identified plasma serpins, including plasminogen activator inhibitor-1 (PAI-1), as potential inhibitors [31,32]. Nonetheless, the same group reported that scFSAP does not bind to PAI-1, leading to some controversy. Some studies have indicated that scFSAP activation increases in the presence of glycosaminoglycans, including hyaluronic acid (hyaluronan) [26,29,31]. As previously noted, hyaluronan is associated with implantation susceptibility, and FSAP activation may be linked to implantation success. There is evidence to support the role of hyaluronan in promoting embryo implantation [33,34,35]. In contrast, one study indicated that hyaluronan enrichment was not beneficial for in vivo fertilization [36]. Furthermore, Fouladi-Nashta et al. [37] described two distinct types of hyaluronic acid—high- and low-molecular-weight forms—with opposing biological functions. They suggested that high-molecular-weight hyaluronan exerts immunosuppressive effects and inhibits differentiation, whereas low-molecular-weight hyaluronan is required for cell proliferation and cell-to-cell interaction during subsequent phases [37,38,39].

In Table 1, the hormone levels are significantly different between the control and case groups. TSH levels are higher in RPL patients. Fumarola et al. reported that pregnancy rates are related to the TSH levels of women; they discovered that high TSH levels (>2.5 mIU/L) result in a low pregnancy rate. Zhou et al. [40] also reported that high TSH level (>3 mIU/L) groups had a resulting low pregnancy rate. But, using an ROC curve, they also reported that it was not enough of a biomarker for pregnancy rates [41]. Seema et al. reported that early pregnancy loss patients have high FSH levels compared with control groups (fertility being measured) [42].

There are limitations in our research. First, case–control studies were conducted only in the Korean population, and studies in other populations will be required. Second, our study sample size is small and does not represent all recurrent pregnancy loss patients. Third, the relationship between the HABP2 genotype and recurrent pregnancy loss is unclear, and a further study for molecular work is required.

## 4. Materials and Methods

### 4.1. Participants

We collected 765 blood samples from 388 female RPL patients and 377 female healthy controls. RPL diagnosis was confirmed through measurements of human chorionic gonadotropin levels, ultrasonography, and physical examination prior to 20 weeks of gestation. Patients were enrolled at the Infertility Medical Center of CHA Bundang Medical Center between March 1999 and February 2012. Control participants were also recruited from CHA Bundang Medical Center. All control participants exhibited a normal 46, XX karyotype, regular menstrual cycles, a history of at least one naturally conceived pregnancy, and no history of pregnancy loss. No RPL patients or control participants had a history of smoking or alcohol use.

Patients with implantation failure due to anatomic, hormonal, chromosomal, infectious, autoimmune, or thrombotic causes were excluded. Anatomic abnormalities were assessed through hysterosalpingography, ultrasonography, computed tomography, hysteroscopy, and magnetic resonance imaging. Hormonal abnormalities, including luteal insufficiency, hyperprolactinemia, or thyroid dysfunction, were excluded after measuring the peripheral blood levels of thyroid-stimulating hormone (TSH), prolactin, follicle-stimulating hormone (FSH), free T4, luteinizing hormone (LH), and progesterone. Lupus anticoagulant and anticardiolipin antibodies were measured to exclude lupus and antiphospholipid syndrome as autoimmune causes of RPL, following previously described methods [43]. The study protocol was reviewed and approved by the institutional review board of CHA Bundang Medical Center (IRB number: 2010-01-123). Written informed consent was obtained from all participants after explanation of the study procedures.

### 4.2. Genotyping

Genomic DNA was extracted from leukocytes using the G-DEX™ II Genomic DNA Extraction Kit (Intron Biotechnology, Seongnam, Republic of Korea), in accordance with the manufacturer’s protocol. Genotyping of six *HABP2* polymorphisms was performed using the TaqMan genotyping assay (Applied Biosystems, Foster City, CA, USA). TaqMan probes were obtained from Applied Biosystems, and genotyping procedures followed the manufacturer’s instructions.

### 4.3. Statistical Analysis

To estimate the relative risk of RPL associated with various genotypes, odds ratios (ORs) and 95% confidence intervals (CIs) were calculated. Statistical significance between the RPL and control groups was assessed using Student’s *t*-test for continuous variables and the χ^2^ test for categorical variables. Multivariate analyses were conducted using logistic regression to adjust for age as a potential confounder. A *p*-value < 0.05 was considered statistically significant. Multiple hypothesis testing was performed using GraphPad Prism 4.0 (GraphPad Software, Inc., San Diego, CA, USA), StatsDirect statistical software Version 2.4.4 (StatsDirect Ltd., Altrincham, UK), MedCalc (Version 7.4 for Windows; MedCalc, Ostend, Belgium), and R software (version 4.5.1; R Foundation for Statistical Computing, Vienna, Austria).

## 5. Conclusions

This study examined the relationships of six *HABP2* polymorphisms with RPL. The rs2240879 CC genotype was associated with an increased RPL risk in Korean women. The rs3832698 variant was associated with coagulation parameters, particularly PT. These findings suggest that *HABP2* represents a biomarker for RPL risk. Further studies are needed to determine whether *HABP2* genotypes are functionally associated with RPL in vitro and in vivo. This study had several potential limitations. First, HABP2’s effect on RPL is unclear; functional studies of SNP’s effect on RPL should be followed. Second, the study population was restricted only to the Korean population. And the current sample size may not fully represent the broader population of RPL patients, highlighting the need for larger cohort studies.

## Figures and Tables

**Table 1:** Baseline characteristics between RPL and controls.

Characteristic	Control (*n* = 377)	RPL (*n* = 388)	*p*
Age	32.920 ± 4.478	32.943 ± 4.132	0.942
PLT	239.571 ± 64.886	245.903 ± 63.476	0.225
PT (s)	10.787 ± 1.713	11.425 ± 1.427	**<0.0001**
aPTT (s)	29.465 ± 3.675	32.017 ± 4.408	**<0.0001**
Folate	14.111 ± 9.886	14.888 ± 13.489	0.971
Homocysteine	7.403 ± 5.127	6.910 ± 2.058	0.845
Total cholesterol	211.293 ± 57.586	187.624 ± 47.627	**<0.0001**
Uric acid	3.832 ± 0.996	3.824 ± 0.860	0.985
BUN	8.946 ± 3.076	10.338 ± 6.334	**<0.0001**
Creatinine	0.638 ± 0.155	0.728 ± 0.122	**<0.0001**
FSH	8.023 ± 2.809	7.800 ± 11.615	**<0.0001**
LH	9.649 ± 17.178	6.355 ± 12.066	0.547
E2	25.818 ± 14.810	50.618 ± 121.261	**0.0003**
TSH	1.533 ± 1.015	2.189 ± 1.511	**<0.0001**
LDL	123.773 ± 40.777	107.760 ± 34.132	0.062
HDL	71.798 ± 21.287	60.387 ± 15.455	**0.002**
Triglycerides	176.000 ± 109.261	186.393 ± 147.057	0.589
Glucose	100.081 ± 25.755	96.682 ± 13.866	0.721
FBS	83.861 ± 20.075	95.134 ± 17.043	**<0.0001**
WBC	7.927 ± 2.503	7.386 ± 2.851	**0.0003**
RBC	4.149 ± 0.394	4.205 ± 0.437	0.103
Hemoglobin	12.423 ± 1.965	12.563 ± 1.363	**0.022**
CD56 NK cell	N/A	17.925 ± 7.911	N/A

Note: RPL, recurrent pregnancy loss; PLT, platelet; PT, prothrombin time; aPTT, activated partial thromboplastin time; BUN, blood urea nitrogen; FSH, follicle-stimulating hormone; LH, luteinizing hormone; E2, estradiol; TSH, thyroid-stimulating hormone; LDL, low-density lipoprotein; HDL, high-density lipoprotein; FBS, fasting blood sugar; WBC, white blood cell; RBC, red blood cell; N/A, not applicable.

**Table 2:** Comparison of genotype frequencies and AOR values of *HABP2* polymorphisms between the RPL and control participants.

rs Number	Control	RPL	COR	*p*	AOR (95% CI)	*p*	PL>3	AOR (95% CI)	*p*
rs3832698									
AA	186 (49.3)	173 (44.6)	1.000 (reference)		1.000 (reference)		90 (42.5)	1.000 (reference)	
A del	149 (39.5)	163 (42.0)	1.176 (0.868–1.594)	0.295	1.170 (0.863–1.586)	0.478	91 (42.9)	0.253 (0.075–0.863)	0.039
del del	42 (11.1)	52 (13.4)	1.331 (0.843–2.101)	0.218	1.332 (0.844–2.102)	0.425	31 (14.6)	0.272 (0.058–1.280)	0.267
Dominant			1.210 (0.911–1.608)	0.188	1.205 (0.906–1.601)	0.438		0.272 (0.087–0.848)	0.054
Recessive			1.234 (0.800–1.905)	0.340	1.238 (0.802–1.911)	0.625		0.538 (0.142–2.045)	0.608
HWE-*P*	0.148	0.172							
rs10885478									
GG	319 (84.6)	326 (84.0)	1.000 (reference)		1.000 (reference)		177 (83.5)	1.000 (reference)	
GA	56 (14.9)	54 (13.9)	0.944 (0.630–1.414)	0.778	0.947 (0.632–1.420)	0.961	31 (14.6)	0.599 (0.135–2.657)	0.752
AA	2 (0.5)	8 (2.1)	3.914 (0.825–18.574)	0.055	3.949 (0.832–18.750)	0.150	4 (1.9)	N/A	N/A
Dominant			1.046 (0.708–1.545)	0.821	1.050 (0.711–1.551)	0.968		0.787 (0.187–3.307)	0.856
Recessive			3.947 (0.833–18.711)	0.053	3.965 (0.836–18.803)	0.153		N/A	N/A
HWE-*P*	0.785	0.003							
rs932650									
TT	189 (50.1)	175 (45.1)	1.000 (reference)		1.000 (reference)		89 (42.0)	1.000 (reference)	
TC	149 (39.5)	163 (42.0)	1.182 (0.873–1.599)	0.280	1.175 (0.868–1.591)	0.507	94 (44.3)	0.273 (0.079–0.946)	0.053
CC	39 (10.3)	50 (12.9)	1.385 (0.868–2.208)	0.170	1.382 (0.867–2.205)	0.230	29 (13.7)	0.294 (0.062–1.386)	0.314
Dominant			1.224 (0.921–1.626)	0.164	1.218 (0.917–1.619)	0.396		0.300 (0.095–0.943)	0.085
Recessive			1.282 (0.822–2.001)	0.272	1.286 (0.824–2.007)	0.538		0.543 (0.143–2.056)	0.613
HWE-*P*	0.237	0.218							
rs7923349									
GG	189 (50.1)	189 (48.7)	1.000 (reference)		1.000 (reference)		110 (51.9)	1.000 (reference)	
GT	148 (39.3)	164 (42.3)	1.108 (0.821–1.496)	0.502	1.101 (0.815–1.486)	0.720	86 (40.6)	0.952 (0.301–3.013)	0.395
TT	40 (10.6)	35 (9.0)	0.875 (0.533–1.438)	0.598	0.859 (0.522–1.413)	0.473	16 (7.5)	0.111 (0.016–0.749)	0.059
Dominant			1.059 (0.797–1.406)	0.694	1.053 (0.793–1.399)	0.936		0.644 (0.233–1.781)	0.629
Recessive			0.835 (0.518–1.347)	0.460	0.837 (0.519–1.350)	0.763		0.112 (0.018–0.700)	0.046
HWE-*P*	0.177	0.946							
rs1157916									
GG	264 (70.0)	260 (67.0)	1.000 (reference)		1.000 (reference)		142 (67.0)	1.000 (reference)	
GA	102 (27.1)	115 (29.6)	1.145 (0.834–1.572)	0.403	1.154 (0.840–1.587)	0.659	66 (31.1)	1.303 (0.412–4.119)	0.862
AA	11 (2.9)	13 (3.4)	1.200 (0.528–2.727)	0.663	1.206 (0.530–2.744)	0.904	4 (1.9)	0.687 (0.056–8.399)	0.905
Dominant			1.150 (0.847–1.561)	0.369	1.155 (0.850–1.568)	0.653		1.204 (0.406–3.571)	0.852
Recessive			1.154 (0.510–2.608)	0.731	1.159 (0.512–2.622)	0.937		0.647 (0.054–7.740)	0.852
HWE-*P*	0.763	0.948							
rs2240879									
TT	258 (68.4)	276 (71.1)	1.000 (reference)		1.000 (reference)		142 (67.0)	1.000 (reference)	
TC	114 (30.2)	94 (24.2)	0.771 (0.559–1.063)	0.112	0.774 (0.561–1.067)	0.293	59 (27.8)	0.855 (0.295–2.476)	0.866
CC	5 (1.3)	18 (4.6)	3.365 (1.232–9.196)	0.010	3.424 (1.252–9.369)	0.028	11 (5.2)	N/A	N/A
Dominant			0.880 (0.646–1.198)	0.416	0.883 (0.648–1.203)	0.731		0.941 (0.328–2.702)	0.896
Recessive			3.620 (1.330–9.851)	0.006	3.642 (1.337–9.918)	0.021		N/A	N/A
HWE-*P*	0.163	0.010							

Note: RPL, recurrent pregnancy loss; COR, crude odds ratio; AOR, adjusted odds ratio; CI, confidence interval; PL, pregnancy loss; HWE, Hardy–Weinberg equilibrium; AOR was adjusted by age.

**Table 3:** Summary of genotype combination analysis of *HABP2* polymorphisms in RPL and control participants.

Genotype Combination	Controls	RPL	AOR (95% CI)	*p*
rs7923349 G>T/rs2240879 T>C				
GG/TT	121 (32.1)	130 (33.5)	1.000 (reference)	
GG/TC	65 (17.2)	50 (12.9)	0.709 (0.454–1.108)	0.074
GG/CC	3 (0.8)	9 (2.3)	3.066 (0.801–11.734)	**0.043**
GT/TT	108 (28.6)	118 (30.4)	1.006 (0.702–1.443)	0.699
GT/TC	39 (10.3)	39 (10.1)	0.930 (0.559–1.547)	0.725
GT/CC	1 (0.3)	7 (1.8)	6.869 (0.827–57.058)	**0.025**
TT/TT	29 (7.7)	28 (7.2)	0.878 (0.492–1.565)	0.543
TT/TC	10 (2.7)	5 (1.3)	0.448 (0.148–1.358)	0.135
TT/CC	1 (0.3)	2 (0.5)	1.877 (0.168–20.979)	0.203
rs2240879 T>C/rs1157916 G>A				
TT/GG	165 (43.8)	173 (44.6)	1.000 (reference)	
TT/GA	94 (24.9)	70 (18.0)	0.716 (0.491–1.043)	0.191
TT/AA	5 (1.3)	17 (4.4)	3.310 (1.192–9.193)	**0.038**
TC/GG	83 (22.0)	90 (23.2)	1.039 (0.718–1.502)	0.975
TC/GA	19 (5.0)	24 (6.2)	1.199 (0.632–2.273)	0.632
TC/AA	0 (0.0)	1 (0.3)	N/A	N/A
CC/GG	10 (2.7)	13 (3.4)	1.279 (0.544–3.007)	0.562
CC/GA	1 (0.3)	0 (0.0)	N/A	N/A
CC/AA	0 (0.0)	0 (0.0)	N/A	N/A

Note: RPL, recurrent pregnancy loss; AOR, adjust odds ratio; CI, confidence interval, adjusted by age.

**Table 4:** Summary of haplotype analysis of the *HABP2*-related polymorphisms associated with increased RPL susceptibility.

Haplotype	Controls (754)	RPL (776)	Combined (1530)	OR (95% CI)	*p*
rs3832698 A>del/rs10885478 G>A/rs932650 T>C/rs7923349 G>T/rs1157916 G>A/rs2240879 T>C					
A-G-T-G-G-T	0.3291	0.3130	0.3223	1.000 (reference)	
A-G-C-T-G-T	0.0000	0.0063	0.0042	11.230 (0.617–204.300)	0.031
del-A-C-T-G-T	0.0139	0.0268	0.0198	2.143 (0.989–4.646)	0.024
rs3832698 A>del/rs932650 T>C/rs7923349 G>T/rs1157916 G>A/rs2240879 T>C					
A-T-G-G-T	0.3279	0.3195	0.3237	1.000 (reference)	
A-C-T-G-T	0.0000	0.0072	0.0044	12.950 (0.725–231.200)	0.017
rs3832698 A>del/rs10885478 G>A/rs932650 T>C/rs7923349 G>T/rs2240879 T>C					
A-G-T-G-T	0.3984	0.3741	0.3868	1.000 (reference)	
del-G-C-G-T	0.1195	0.1497	0.1332	1.333 (0.969–1.835)	0.038
del-A-C-T-T	0.0148	0.0279	0.0222	2.069 (0.985–4.344)	0.025
rs3832698 A>del/rs10885478 G>A/rs932650 T>C/rs7923349 G>T/rs1157916 G>A					
A-G-T-G-G	0.4149	0.3927	0.4049	1.000 (reference)	
A-G-C-T-G	0.0000	0.0060	0.0041	11.290 (0.621–205.200)	0.030
rs10885478 G>A/rs7923349 G>T/rs1157916 G>A/rs2240879 T>C					
G-G-G-T	0.4092	0.4078	0.4088	1.000 (reference)	
A-T-G-T	0.0135	0.0262	0.0204	1.956 (0.901–4.246)	0.042
rs10885478 G>A/rs932650 T>C/rs7923349 G>T/rs2240879 T>C					
G-T-G-T	0.4011	0.3814	0.3919	1.000 (reference)	
A-C-T-T	0.0144	0.0307	0.0236	2.226 (1.071–4.627)	0.014
rs3832698 A>del/rs7923349 G>T/rs1157916 G>A/rs2240879 T>C					
A-G-G-T	0.3364	0.3222	0.3289	1.000 (reference)	
del-G-A-T	0.0532	0.0734	0.0629	1.448 (0.932–2.249)	0.049
rs3832698 A>del/rs932650 T>C/rs1157916 G>A/rs2240879 T>C					
A-T-G-T	0.4929	0.4538	0.4731	1.000 (reference)	
del-C-A-T	0.0677	0.0870	0.0792	1.409 (0.953–2.084)	0.042
rs3832698 A>del/rs932650 T>C/rs7923349 G>T/rs1157916 G>A					
A-T-G-G	0.4129	0.3978	0.4051	1.000 (reference)	
A-C-T-G	0.0000	0.0069	0.0039	11.070 (0.609–201.200)	0.032
rs3832698 A>del/rs10885478 G>A/rs7923349 G>T/rs2240879 T>C					
A-G-G-T	0.4083	0.3824	0.3955	1.000 (reference)	
del-G-G-T	0.1236	0.1521	0.1374	1.316 (0.960–1.803)	0.044
del-A-T-T	0.0148	0.0274	0.0217	1.980 (0.938–4.178)	0.034
rs3832698 A>del/rs10885478 G>A/rs932650 T>C/rs7923349 G>T					
A-G-T-G	0.4852	0.4583	0.4720	1.000 (reference)	
del-G-C-G	0.1458	0.1775	0.1611	1.290 (0.965–1.724)	0.043
del-A-T-T	0.0000	0.0032	0.0015	0.881 (0.323–2.507)	0.0001
rs10885478 G>A/rs7923349 G>T/rs2240879 T>C					
G-G-T	0.5315	0.5345	0.5329	1.000 (reference)	
A-T-T	0.0147	0.0328	0.0248	2.196 (1.066–4.523)	0.015
rs3832698 A>del/rs932650 T>C/rs2240879 T>C					
A-T-T	0.5784	0.5359	0.5563	1.000 (reference)	
del-C-T	0.2309	0.2634	0.2477	1.229 (0.964–1.567)	0.048
rs3832698 A>del/rs10885478 G>A/rs2240879 T>C					
A-G-T	0.5903	0.5449	0.5666	1.000 (reference)	
del-G-T	0.1901	0.2178	0.2044	1.327 (1.020–1.726)	0.017
rs3832698 A>del/rs2240879 T>C					
A-T	0.5895	0.5497	0.5687	1.000 (reference)	
del-T	0.2460	0.2828	0.2653	1.231 (0.972–1.559)	0.043

Note: HABP2, hyaluronan-binding protein 2; RPL, recurrent pregnancy loss; OR, odds ratio; CI, confidence interval.

**Table 5:** Synergistic effect of *HABP2* polymorphisms with clinical risk factors.

Characteristics	rs3832698 A>del				rs10885478 G>A				rs932650 T>C			
	AA AOR (95% CI)	*p*	A del + del del AOR (95% CI)	*p*	GG AOR (95% CI)	*p*	GA + AA AOR (95% CI)	*p*	TT AOR (95% CI)	*p*	TC + CC AOR (95% CI)	*p*
Age												
<34	1.000 (reference)		1.448 (0.908–2.310)	0.138	1.000 (reference)		0.894 (0.483–1.658)	0.434	1.000 (reference)		1.250 (0.784–1.992)	0.298
≥34	0.914 (0.492–1.698)	0.918	0.600 (0.307–1.172)	0.078	0.593 (0.365–0.963)	0.099	0.656 (0.297–1.450)	0.468	0.925 (0.502–1.706)	0.838	0.530 (0.267–1.050)	**0.041**
FSH												
<7.93	1.000 (reference)		1.167 (0.861–1.582)	0.603	1.000 (reference)		0.960 (0.623–1.481)	0.976	1.000 (reference)		1.155 (0.852–1.564)	0.645
≥7.93	0.722 (0.351–1.483)	0.641	1.136 (0.654–1.980)	0.536	0.799 (0.486–1.313)	0.645	1.330 (0.581–3.048)	0.718	0.651 (0.315–1.348)	0.437	1.248 (0.718–2.167)	0.693
E2												
<40.9	1.000 (reference)		1.156 (0.858–1.556)	0.626	1.000 (reference)		0.958 (0.631–1.454)	0.967	1.000 (reference)		1.139 (0.846–1.533)	0.684
≥40.9	3.690 (1.435–9.484)	**0.013**	4.762 (2.133–10.630)	**<0.001**	3.817 (1.916–7.605)	**<0.001**	4.659 (1.314–16.521)	**0.025**	3.366 (1.292–8.773)	**0.026**	5.107 (2.299–11.347)	**<0.001**
LH												
<33.38	1.000 (reference)		1.058 (0.779–1.438)	0.896	1.000 (reference)		0.817 (0.538–1.240)	0.609	1.000 (reference)		1.049 (0.772–1.424)	0.913
≥33.38	0.210 (0.101–0.440)	**<0.001**	0.631 (0.344–1.158)	0.260	0.267 (0.158–0.454)		1.664 (0.560–4.942)	0.634	0.192 (0.092–0.400)	**<0.001**	0.659 (0.355–1.221)	0.194
TSH												
<1.97	1.000 (reference)		1.184 (0.864–1.622)	0.428	1.000 (reference)	**<0.001**	0.940 (0.610–1.449)	0.715	1.000 (reference)		1.145 (0.836–1.568)	0.521
≥1.97	3.520 (1.898–6.530)	**<0.001**	4.140 (2.324–7.375)	**<0.001**	3.132 (2.003–4.899)	**<0.001**	7.108 (2.069–24.421)	**0.001**	3.089 (1.680–5.680)	**0.001**	4.485 (2.496–8.056)	**<0.001**
PT												
<11.8	1.000 (reference)		1.019 (0.742–1.400)	0.986	1.000 (reference)		0.927 (0.588–1.462)	0.941	1.000 (reference)		1.012 (0.737–1.389)	0.990
≥11.8	4.734 (2.390–9.377)	**<0.001**	9.176 (4.686–17.971)	**<0.001**	7.128 (4.135–12.289)	**<0.001**	5.471 (2.207–13.564)	**<0.001**	4.257 (2.196–8.254)	**<0.001**	10.062 (5.013–20.199)	**<0.001**
aPTT												
>27.5	1.000 (reference)		1.205 (0.882–1.648)	0.419	1.000 (reference)		0.967 (0.633–1.477)	0.823	1.000 (reference)		1.224 (0.895–1.672)	0.374
≤27.5	0.412 (0.223–0.760)	**0.013**	0.568 (0.335–0.964)	0.062	0.426 (0.277–0.655)	**<0.001**	0.596 (0.223–1.588)	0.578	0.420 (0.230–0.767)	**0.012**	0.562 (0.329–0.958)	0.097
PLT												
<242.4	1.000 (reference)		1.375 (0.954–1.981)	0.218	1.000 (reference)		1.366 (0.810–2.303)	0.472	1.000 (reference)		1.404 (0.975–2.023)	0.178
≥242.4	1.022 (0.668–1.563)	0.951	1.013 (0.672–1.527)	0.774	0.943 (0.686–1.297)	0.897	0.706 (0.393–1.267)	0.339	1.042 (0.682–1.591)	0.849	1.022 (0.680–1.538)	0.570
Characteristics	rs7923349 G>T				rs1157916 G>A				rs2240879 T>C			
	GG AOR (95% CI)	*p*	GT + TT AOR (95% CI)	*p*	GG AOR (95% CI)	*p*	GA + AA AOR (95% CI)	*p*	TT AOR (95% CI)	*p*	TC + CC AOR (95% CI)	*p*
Age												
<34	1.000 (reference)		1.426 (0.895–2.273)	0.151	1.000 (reference)		0.849 (0.510–1.415)	0.380	1.000 (reference)		1.040 (0.629-.7184)	0.457
≥34	0.600 (0.309–1.163)	0.064	0.879 (0.484–1.597)	0.908	0.497 (0.281–0.878)	**0.050**	1.116 (0.596–2.090)	0.922	0.742 (0.438–1.256)	0.484	0.446 (0.223–0.890)	0.070
FSH												
<7.93	1.000 (reference)		1.086 (0.802–1.472)	0.860	1.000 (reference)		1.169 (0.842–1.624)	0.643	1.000 (reference)		0.885 (0.636–1.232)	0.763
≥7.93	0.910 (0.478–1.734)	0.194	0.852 (0.470–1.545)	0.338	0.966 (0.568–1.642)	0.930	1.033 (0.500–2.133)	0.947	0.910 (0.540–1.534)	0.848	0.823 (0.392–1.729)	0.806
E2												
<40.9	1.000 (reference)		1.015 (0.754–1.367)	0.982	1.000 (reference)		1.173 (0.852–1.616)	0.611	1.000 (reference)		0.865 (0.625–1.197)	0.671
≥40.9	2.560 (1.186–5.522)	**0.009**	7.285 (2.493–21.289)	**<0.001**	4.163 (2.016–8.597)	**<0.001**	4.685 (1.537–14.281)	**0.009**	4.025 (1.890–8.574)	**<0.001**	3.527 (1.281–9.710)	**0.029**
LH												
<33.38	1.000 (reference)		1.004 (0.740–1.363)	0.956	1.000 (reference)		1.056 (0.759–1.470)	0.907	1.000 (reference)		0.896 (0.642–1.251)	0.776
≥33.38	0.318 (0.163–0.624)	**<0.001**	0.486 (0.262–0.900)	0.031	0.279 (0.156–0.500)	**<0.001**	0.665 (0.322–1.376)	0.466	0.381 (0.220–0.662)	**0.002**	0.345 (0.161–0.740)	**0.016**
TSH												
<1.97	1.000 (reference)		1.238 (0.904–1.696)	0.307	1.000 (reference)		1.278 (0.911–1.791)	0.271	1.000 (reference)		0.716 (0.504–1.017)	0.128
≥1.97	4.548 (2.557–8.090)	**<0.001**	3.199 (1.710–5.988)	<0.001	4.192 (2.503–7.023)	**<0.001**	3.188 (1.580–6.431)	**0.002**	2.719 (1.636–4.521)	**<0.001**	4.345 (2.120–8.905)	**<0.001**
PT												
<11.8	1.000 (reference)		1.130 (0.823–1.553)	0.746	1.000 (reference)		1.271 (0.909–1.777)	0.372	1.000 (reference)		0.784 (0.553–1.112)	0.389
≥11.8	7.926 (4.005–15.687)	**<0.001**	6.561 (3.377–12.748)	**<0.001**	6.340 (3.747–10.727)	**<0.001**	15.385 (4.556–51.958)	**<0.001**	5.395 (3.125–9.314)	**<0.001**	9.573 (3.701–24.760)	**<0.001**
aPTT												
>27.5	1.000 (reference)		1.151 (0.843–1.573)	0.563	1.000 (reference)		1.204 (0.860–1.6865)	0.464	1.000 (reference)		0.879 (0.628–1.230)	0.628
≤27.5	0.583 (0.330–1.029)	**0.034**	0.409 (0.233–0.717)	**0.001**	0.490 (0.306–0.783)	**0.009**	0.432 (0.211–0.882)	0.057	0.472 (0.299–0.746)	**0.004**	0.322 (0.145–0.711)	**0.012**
PLT												
<242.4	1.000 (reference)		1.090 (0.758–1.568)	0.846	1.000 (reference)		1.060 (0.717–1.569)	0.903	1.000 (reference)		0.996 (0.671–1.478)	0.943
≥242.4	0.884 (0.583–1.342)	0.171	0.894 (0.594–1.347)	0.856	0.801 (0.563–1.140)	0.439	1.086 (0.680–1.733)	0.609	0.942 (0.665–1.334)	0.852	0.682 (0.425–1.096)	0.282

Note: HABP2, hyaluronan-binding protein 2; AOR, adjusted odds ratio; CI, confidence interval; PT, prothrombin time; aPTT, activated partial thromboplastin time; FSH, follicle-stimulating hormone; LH, luteinizing hormone; E2, estradiol; TSH, thyroid-stimulation hormone, PLT, platelet; AOR was adjusted by age.

**Table 6:** Summary of one-way analysis of variance between clinical parameters and each *HABP2* polymorphism in total participants.

rs Number	Platelet	PT (s)	aPTT (s)	Creatinine	LDL	HDL	Triglycerides
rs3832698							
AA	244.93 ± 65.45	11.10 ± 0.97	30.72 ± 4.20	0.68 ± 0.15	121.65 ± 41.32	71.08 ± 22.23	164.07 ± 116.99
A del	237.75 ± 61.19	11.08 ± 0.91	30.73 ± 4.29	0.69 ± 0.15	121.95 ± 39.88	70.15 ± 21.13	188.72 ± 125.12
del del	249.23 ± 69.89	11.45 ± 1.25	31.71 ± 4.49	0.67 ± 0.13	126.10 ± 41.22	63.38 ± 14.48	216.41 ± 155.32
*p*	0.269	**0.031**	0.241	0.607	0.902	0.267	0.112
rs10885478							
GG	242.32 ± 64.58	11.09 ± 0.99	30.82 ± 4.30	0.68 ± 0.14	122.64 ± 41.42	70.73 ± 21.64	173.80 ± 119.87
GA	242.74 ± 63.98	11.32 ± 0.92	30.75 ± 4.02	0.71 ± 0.15	118.09 ± 35.02	65.40 ± 18.13	226.14 ± 149.18
AA	245.25 ± 54.85	12.01 ± 1.26	34.44 ± 5.17	0.70 ± 0.13	105.00 ± 65.05	72.77 ± 14.78	97.20 ± 74.60
*p*	0.991	**0.029**	0.167	0.328	0.704	0.333	**0.019**
rs932650							
TT	244.82 ± 66.69	11.11 ± 0.97	30.61 ± 4.19	0.68 ± 0.15	121.67 ± 41.79	70.96 ± 22.68	163.29 ± 116.97
CT	237.95 ± 60.67	11.09 ± 0.97	30.79 ± 4.25	0.69 ± 0.15	123.27 ± 39.76	70.57 ± 21.23	191.93 ± 124.00
CC	249.01 ± 67.00	11.40 ± 1.10	31.92 ± 4.64	0.68 ± 0.14	119.48 ± 39.72	63.91 ± 13.68	206.07 ± 159.25
*p*	0.300	0.090	0.100	0.645	0.915	0.421	0.083
rs7923349							
GG	243.00 ± 63.22	11.14 ± 0.95	31.06 ± 4.40	0.69 ± 0.15	125.50 ± 40.68	70.50 ± 22.03	178.62 ± 132.23
GT	240.77 ± 62.63	11.06 ± 0.96	30.44 ± 4.10	0.67 ± 0.14	117.37 ± 38.74	70.69 ± 21.25	182.28 ± 119.19
TT	246.28 ± 76.27	11.44 ± 1.30	31.50 ± 4.31	0.72 ± 0.15	125.56 ± 46.54	66.13 ± 18.88	178.00 ± 117.05
*p*	0.817	0.081	0.167	0.158	0.335	0.582	0.968
rs1157916							
GG	241.32 ± 65.54	11.18 ± 1.04	30.92 ± 4.39	0.69 ± 0.14	122.60 ± 40.47	72.17 ± 22.10	177.95 ± 123.44
GA	244.88 ± 62.36	11.07 ± 0.86	30.65 ± 4.04	0.68 ± 0.14	118.65 ± 37.51	65.13 ± 17.70	189.62 ± 133.51
AA	245.91 ± 54.15	10.69 ± 0.75	30.76 ± 3.96	0.71 ± 0.19	139.57 ± 66.77	70.50 ± 28.46	154.36 ± 108.16
*p*	0.808	0.092	0.817	0.719	0.41	0.160	0.618
rs2240879							
TT	242.30 ± 62.28	11.11 ± 1.01	30.69 ± 4.26	0.68 ± 0.14	119.43 ± 39.18	69.81 ± 21.13	181.16 ± 124.91
TC	243.52 ± 68.43	11.15 ± 0.93	31.05 ± 4.34	0.68 ± 0.16	129.53 ± 43.26	70.41 ± 21.27	168.57 ± 118.81
CC	236.19 ± 72.91	11.35 ± 0.99	32.08 ± 4.16	0.71 ± 0.14	115.60 ± 41.46	79.90 ± 31.87	277.50 ± 176.29
*p*	0.884	0.548	0.283	0.739	0.229	0.579	0.061

Note: PT, prothrombin time; aPTT, activated partial thromboplastin time; LDL, low-density lipoprotein; HDL, high-density lipoprotein; AOR was adjusted by age.

**Table 7:** Summary of one-way analysis of variance between clinical parameters and each *HABP2* polymorphism in RPL patients.

rs Number	PLT	PT (s)	aPTT (s)	Creatinine	LDL	HDL	Triglycerides
rs3832698							
AA	255.03 ± 68.78	11.47 ± 0.87	31.84 ± 4.34	0.72 ± 0.13	107.27 ± 34.30	59.52 ± 18.70	166.19 ± 142.31
A del	236.75 ± 59.38	11.49 ± 0.80	31.85 ± 4.49	0.74 ± 0.12	111.78 ± 30.89	57.85 ± 13.27	193.28 ± 140.97
del del	245.46 ± 54.18	11.94 ± 1.26	33.20 ± 4.27	0.72 ± 0.09	129.67 ± 62.78	69.46 ± 11.45	234.50 ± 189.19
*p*	0.081	**0.020**	0.234	0.583	0.629	0.337	0.317
rs10885478							
GG	247.94 ± 65.61	11.52 ± 0.91	32.06 ± 4.49	0.72 ± 0.12	110.64 ± 32.64	60.17 ± 16.34	181.78 ± 146.90
GA	237.07 ± 53.01	11.60 ± 0.86	31.43 ± 3.83	0.76 ± 0.12	120.00 ± 56.24	53.21 ± 8.96	231.67 ± 150.31
AA	228.00 ± 39.67	12.31 ± 1.23	35.90 ± 4.63	0.73 ± 0.10	105.00 ± 65.05	72.77 ± 14.78	92.25 ± 85.18
*p*	0.472	0.206	0.144	0.261	0.892	0.192	0.180
rs932650							
TT	255.45 ± 69.04	11.49 ± 0.87	31.70 ± 4.34	0.72 ± 0.13	107.27 ± 34.30	58.35 ± 18.66	166.06 ± 142.41
CT	235.55 ± 58.66	11.50 ± 0.90	31.90 ± 4.44	0.74 ± 0.13	111.78 ± 30.89	58.82 ± 13.67	195.07 ± 139.74
CC	248.89 ± 55.47	11.84 ± 1.05	33.43 ± 4.38	0.72 ± 0.09	129.67 ± 62.78	69.46 ± 11.45	226.50 ± 195.03
*p*	**0.049**	0.114	0.112	0.481	0.629	0.353	0.370
rs7923349							
GG	243.60 ± 65.11	11.54 ± 0.85	32.10 ± 4.55	0.74 ± 0.13	116.08 ± 37.47	58.93 ± 13.90	187.03 ± 164.15
GT	250.52 ± 60.14	11.48 ± 0.90	31.78 ± 4.26	0.72 ± 0.11	106.85 ± 36.30	61.33 ± 18.34	187.20 ± 126.41
TT	236.74 ± 70.55	11.90 ± 1.27	32.73 ± 4.36	0.75 ± 0.09	106.00 ± 0.00	56.65 ± 2.62	176.63 ± 152.16
*p*	0.531	0.144	0.617	0.312	0.809	0.856	0.982
rs1157916							
GG	244.05 ± 68.55	11.62 ± 0.96	32.16 ± 4.54	0.74 ± 0.12	99.06 ± 26.77	61.03 ± 17.12	171.86 ± 133.22
GA	247.13 ± 51.10	11.44 ± 0.74	31.62 ± 4.12	0.71 ± 0.13	134.10 ± 39.14	58.74 ± 13.07	223.15 ± 172.40
AA	269.18 ± 48.52	10.81 ± 0.86	32.28 ± 4.32	0.67 ± 0.14	78.00 ± 0.00	53.45 ± 18.17	161.00 ± 161.57
*p*	0.435	**0.012**	0.654	0.091	**0.026**	0.767	0.223
rs2240879							
TT	245.63 ± 61.77	11.51 ± 0.92	31.84 ± 4.46	0.72 ± 0.12	116.16 ± 38.62	60.20 ± 16.08	189.47 ± 146.98
TC	245.83 ± 67.62	11.60 ± 0.94	32.32 ± 4.37	0.76 ± 0.13	99.63 ± 26.35	59.18 ± 15.08	154.47 ± 128.13
CC	249.56 ± 70.81	11.64 ± 0.79	32.87 ± 4.07	0.74 ± 0.12	0.00 ± 0.00	0.00 ± 0.00	316.17 ± 189.82
*p*	0.972	0.684	0.535	0.050	0.281	0.852	**0.043**

Note: PLT, platelet; PT, prothrombin time; aPTT, activated partial thromboplastin time; LDL, low-density lipoprotein; HDL, high-density lipoprotein.

**Table 8:** Summary of one-way analysis of variance between clinical parameters and each *HABP2* polymorphism in controls.

rs Number	PLT	PT (s)	aPTT (s)	Creatinine	LDL	HDL	Triglycerides
rs3832698							
AA	237.23 ± 61.90	10.72 ± 0.92	29.58 ± 3.74	0.65 ± 0.15	124.04 ± 42.06	73.08 ± 22.26	162.92 ± 101.46
A del	238.61 ± 62.91	10.53 ± 0.74	29.25 ± 3.52	0.64 ± 0.16	122.98 ± 40.68	72.83 ± 21.62	185.20 ± 112.26
del del	252.62 ± 82.05	10.74 ± 0.83	29.70 ± 4.02	0.61 ± 0.15	125.47 ± 39.02	61.78 ± 15.02	203.65 ± 131.11
*p*	0.405	0.262	0.767	0.507	0.968	0.098	0.239
rs10885478							
GG	237.79 ± 63.48	10.61 ± 0.84	29.42 ± 3.61	0.64 ± 0.15	124.22 ± 42.27	72.63 ± 21.96	168.84 ± 99.69
GA	247.29 ± 71.79	10.93 ± 0.87	29.80 ± 4.15	0.65 ± 0.17	117.90 ± 33.73	67.98 ± 18.61	222.16 ± 151.35
AA	297.00 ± 77.78	10.80 ± 0.00	28.60 ± 0.00	0.50 ± 0.00	0.00 ± 0.00	0.00 ± 0.00	117.00 ± 0.00
*p*	0.287	0.140	0.840	0.655	0.433	0.255	0.298
rs932650							
TT	236.99 ± 64.00	10.73 ± 0.92	29.54 ± 3.74	0.65 ± 0.15	123.97 ± 42.57	72.94 ± 22.70	161.80 ± 101.52
CT	240.06 ± 62.51	10.51 ± 0.74	29.29 ± 3.47	0.63 ± 0.16	124.48 ± 40.54	73.34 ± 21.79	189.44 ± 110.91
CC	249.14 ± 77.38	10.74 ± 0.81	29.75 ± 4.18	0.62 ± 0.17	117.78 ± 37.03	62.53 ± 14.09	192.44 ± 134.76
*p*	0.587	0.170	0.820	0.658	0.817	0.116	0.224
rs7923349							
GG	242.51 ± 61.81	10.66 ± 0.83	29.82 ± 3.88	0.64 ± 0.16	126.80 ± 41.12	72.90 ± 22.68	173.17 ± 107.27
GT	232.35 ± 63.74	10.58 ± 0.79	28.87 ± 3.28	0.63 ± 0.15	119.08 ± 39.07	72.81 ± 21.38	178.96 ± 114.81
TT	252.22 ± 79.98	10.92 ± 1.16	30.21 ± 3.97	0.68 ± 0.18	126.38 ± 47.36	66.86 ± 19.42	178.79 ± 98.30
*p*	0.183	0.284	0.103	0.316	0.440	0.423	0.940
rs1157916							
GG	239.17 ± 63.14	10.68 ± 0.88	29.53 ± 3.77	0.63 ± 0.15	125.33 ± 40.97	74.13 ± 22.34	181.67 ± 117.44
GA	242.89 ± 71.09	10.59 ± 0.77	29.40 ± 3.59	0.65 ± 0.15	115.84 ± 36.88	66.67 ± 18.41	163.12 ± 85.28
AA	220.30 ± 50.10	10.52 ± 0.57	28.66 ± 2.24	0.76 ± 0.25	149.83 ± 66.82	76.18 ± 30.22	150.57 ± 79.99
*p*	0.573	0.749	0.801	**0.043**	0.097	0.156	0.856
rs2240879							
TT	239.48 ± 62.71	10.65 ± 0.91	29.33 ± 3.57	0.65 ± 0.16	119.88 ± 39.38	71.74 ± 21.53	175.83 ± 108.73
TC	242.04 ± 69.22	10.69 ± 0.67	29.82 ± 3.97	0.62 ± 0.15	133.80 ± 43.67	72.78 ± 21.72	176.93 ± 113.33
CC	193.40 ± 69.07	10.13 ± 0.88	28.75 ± 2.98	0.60 ± 0.18	115.60 ± 41.46	79.90 ± 31.87	161.50 ± 38.89
*p*	0.263	0.439	0.605	0.389	0.091	0.695	0.981

Note: PLT, platelet; PT, prothrombin time; aPTT, activated partial thromboplastin time; LDL, low-density lipoprotein; HDL, high-density lipoprotein.

## Data Availability

The original contributions presented in this study are included in the article. Further inquiries can be directed to the corresponding authors.
